# Supplementary material for: Metrics for comparing neuronal tree shapes based on persistent homology
Source: PLoS One. 2017 Aug 15;12(8):e0182184. doi: 10.1371/journal.pone.0182184 (PMC5557505; doi:10.1371/journal.pone.0182184)
Supplement: S1 File — (PDF) [file pone.0182184.s001.pdf]

Supplement S1 :

Stability of persistence-based signatures

Given two neuron trees T_1 and T_2, in (Step 1) we first map them to their respective persistence diagrams D_1 and D_2 induced by some descriptor function(s). In (Step 2), we further vectorize these persistence diagrams into persistence feature vectors, say V_1 and V_2 respectively. It is desirable that such a feature generation and vectorization process is *stable* in the sense that "small perturbations" in input neuronal trees and in the induced descriptor functions should only cause small changes in the distances between them. Making such a stability statement precise is not trivial, depending also on how "perturbations" are modeled and measured. While we do not yet have a full stability statement for our persistence-based feature vectors, below we discuss some partial results. We separately consider the stability for persistence diagrams (after Step-1) and that of persistence-based feature vectors (after Step-2).

Stability of persistence diagrams. To discuss stability, we first need to measure the distance between two persistence diagrams. Given two persistence diagrams D_1 and D_2 (each of which consists of a set of points in $\mathbb{R}^2$), there is a natural distance measure, the *bottleneck distance* $d_B(D_1, D_2)$ first introduced in [1]. Consider matching points in D_1 with points in D_2 such that each point in D_1 (resp. in D_2) has to be matched, either to a unique point in D_2 (resp. in D_1), or to its nearest neighbor in the diagonal $\mathrm{L} := \{(x, x) \mid x \in \mathbb{R}\}$: The latter case corresponds to treating this feature point as noise, in which case it is matched to a persistent point with zero persistence. See Fig 2C in the main submission for an illustration. Find the optimal correspondence so that the maximum distance between pairs of corresponding points is minimized; $d_B(D_1, D_2)$ equals this smallest possible maximum distance.

More precisely,

$$\text{Set } \widehat{D}_1 := D_1 \cup \mathrm{L} \text{ and } \widehat{D}_2 := D_2 \cup \mathrm{L}, \quad \text{then } \; d_B(D_1, D_2) = \inf_{\gamma: \widehat{D}_1 \to \widehat{D}_2} \sup_{u \in \widehat{D}_1} \|u - \gamma(u)\|_\infty, \qquad (1)$$

where $\|u - v\|_\infty = \max\{|u.x - v.x|, |u.y - v.y|\}$ denotes the L_∞ distance between two points; and γ ranges over all bijections between $\widehat{D}_1$ and $\widehat{D}_2$. It is known that the bottleneck distance $d_B(D_1, D_2)$ can be computed in $O(m^{1.5} \log m)$ time, where m is the total number of points in $D_1 \cup D_2$.

Given two functions $f, g : |T| \to \mathbb{R}$, suppose that g is a perturbation of f with bounded distance in L_∞-norm, that is $\|f - g\|_\infty := \max_{x \in |T|} |f(x) - g(x)|$ measures the amount of perturbation of g from f. The Stability Theorem [1] states that for a function f and its perturbation g, the bottleneck distance between their persistence diagram summaries is bounded from above by the size of the perturbation; that is, $d_B(\mathrm{Dg} f, \mathrm{Dg}\ g) \leq \|f - g\|_\infty$. This result is later generalized to more general persistence modules, and show that the bottleneck distance between two persistent diagrams is bounded by the so-called interleaving distance between the corresponding persistence modules that generate them [2, 3].

In our setting, given two neuron trees T_1 and T_2 with descriptor functions $f_1 : |T_1| \to \mathbb{R}$ and $f_2 : |T_2| \to \mathbb{R}$, we cannot directly compare these two descriptor functions since they are defined on different domains (T_1 and T_2, respectively). We instead use the so-called *functional distortion distance* [4] $d_{FD}(f_1, f_2)$ to measure how different the functions f_1 and f_2 are. Intuitively, d_{FD} considers all pairs of mappings between T_1 and T_2 as a way to align them, say $\phi : |T_1| \to |T_2|$ and $\psi : |T_2| \to |T_1|$ to align T_1 to T_2 (via ϕ) as well as align T_2 to T_1 (via ψ). It then compares f_1 and f_2 composited with these maps so that they are then defined on a common domain. Each such pair

of maps (alignment) (ϕ, ψ) will give a cost, measuring how well f_1 and f_2 are aligned under these two maps, and $d_{FD}(f_1, f_2)$ returns the minimum cost under all possible such alignments (pairs of maps). We refer the readers to [4] see the formal definition. It follows from results of [4] that $d_B(\mathrm{Dg} f_1, \mathrm{Dg} f_2) \leq d_{FD}(f_1, f_2)$.

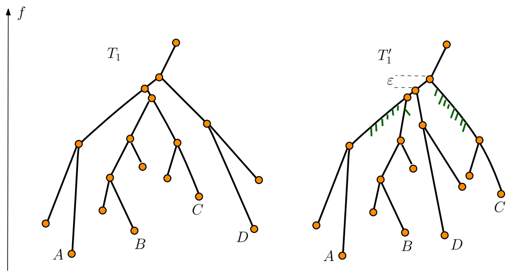

Figure 1: T'_1 is a noisy version of T_1: there could be local combinatorial changes such as the subtrees B, C and D merge at slightly different height, and there could also be spurious noisy branches. However, such changes do not perturb the tree metric much: in this specific example, the metric distortion is bounded by ε. As a result, their corresponding persistence diagram summaries are also close with $d_B(D, D') \leq \varepsilon$.

This stability result applies to any descriptor functions. For example, suppose we consider the Euclidean distance functions f_1 and f_2 on the two trees in Figure 1. Then their persistence diagram summaries are close (at most ε), despite that there are noisy branches, as well as combinatorial changes in the tree structures from T_1 to T_2 – Indeed, it is not hard to establish maps $\phi : T_1 \to T_2$ and $\psi : T_2 \to T_1$ and show that the cost of the Euclidean distance function incurred by them is at most ε, thus upper bounds $d_{FD}(T_1, T_2)$ by ε too.

As another example, if we use the geodesic distance to the root as the descriptor function, then by using results from [5], the bottleneck distance between resulting persistence diagrams is stable w.r.t. changes in the input neuron trees as measured by the Gromov-Hausdorff distance between these trees. The Gromov-Hausdorff distance is popular way to measure the level of near-isometry between two metric spaces [6, 7]. The Gromove-Hausdorff distance between the two neuron trees in Figure 1 is at most ε, implying that using the geodesic distance as descriptor functions $f : T_1 \to \mathbb{R}$ and $f' : T'_1 \to \mathbb{R}$, we have $d_B(\mathrm{Dg} f, \mathrm{Dg} f') \leq \varepsilon$ as well.

In our framework, to improve computational efficiency, we vectorize the persistent diagrams describe in (Step 2), and the natural L_p-distance between them are sum-based, instead of max-based (as in bottleneck distance). One can extend the bottleneck distance to the so-called degree p-Wasserstein distance $d_{W,p}(D_1, D_2)$ between two persistence diagrams D_1 and D_2 which we will introduce shortly in Eqn (2). The stability for the Wasserstein distance of persistence diagrams is not as well understood as in the bottleneck distance case (which is in fact the case when $p = \infty$), although there are some results for some special cases [8].

Stability of the persistence feature vectors. We now discuss the stability of feature vectorization step. Specifically, suppose we are given two persistence diagrams D_1 and D_2, with corresponding feature functions $\rho_1 = \rho_{D_1}, \rho_2 = \rho_{D_2} : \mathbb{R} \to \mathbb{R}$ induced from D_1 and D_2 as described in Section 2.2 of the main text.

First, the *degree-p Wasserstein distance between D_1 and D_2*, for $1 \leq p < \infty$, is defined as:

$$d_{W,p}(D_1, D_2) = \inf_{\gamma : \widehat{D}_1 \to \widehat{D}_2} \left(\sum_{u \in \widehat{D}_1} \| u - \gamma(u) \|_\infty^p \right)^{1/p}, \qquad (2)$$

where $\widehat{D}_1 := D_1 \cup \mathrm{L}$ and $\widehat{D}_2 := D_2 \cup \mathrm{L}$ are the persistence diagrams augmented with points in the diagonal L as before.

Theorem 0.1 *The L_1-distance between feature functions ρ_1 and ρ_2 is stable w.r.to the 1-Wasserstein distance between the diagrams D_1 and D_2 generating them. Let Δ denote the largest persistence value of any point in D_1; that is, $\Delta = \max_{\langle u,u'\rangle \in D_1} |u' - u|$. Specifically,*

$$\|\rho_1 - \rho_2\|_1 \leq (1 + \sqrt{\frac{2}{\pi}}\Delta) \cdot d_{W,1}(D_1, D_2).$$

We now prove this theorem. First, we need the following result bounding the distance between two 1-dimensional Gaussians [9].

Lemma 0.2 ([9]) *Given $u \in \mathbb{R}$, let $g_u : \mathbb{R} \to \mathbb{R}$ denote the normalized 1-dimensional Gaussian centered at $u \in \mathbb{R}$: $g_u = \frac{1}{\sigma\sqrt{2\pi}} e^{-(z-u)^2/2\sigma^2}$. For $a, b > 0$ and $u, v \in \mathbb{R}$, we then have:*

$$\|ag_u - bg_v\|_1 \leq |a - b| + \sqrt{\frac{2}{\pi}} \frac{\min\{a, b\}}{\sigma} |u - v|.$$

Now let $\gamma^* : \widehat{D}_1 \to \widehat{D}_2$ denote the optimal bijection to give rise to $d_{W,1}(D_1, D_2)$; that is,

$$d_{W,p}(D_1, D_2) = \sum_{w \in \widehat{D}_1} ||w - \gamma^*(w)||_\infty.$$

Assume w.o.l.g that all persistent points lie above the diagonal in the persistence diagrams D_1 and D_2. Recall by Eqn (4) of the main text, for $i = 1$ or 2, the two feature functions are

$$\rho_i = \rho_{D_i} = \sum_{\langle u,u'\rangle \in D_i} (u' - u) \cdot g_u.$$

Since for all points $(u, u') \in \mathrm{L}$ in the diagonal L, we have that $u = u'$. It then follows that equivalently, we have that

$$\rho_i = \sum_{\langle u,u'\rangle \in \widehat{D}_i} (u' - u) \cdot g_u.$$

Finally, for each point $w = \langle u, u'\rangle \in \widehat{D}_i$, we also denote $w.birth = u$ (the birth time of this

point) and $w.pers = |u' - u|$ (the persistence of this point). We now have:

$$\begin{aligned}
\|\rho_1 - \rho_2\|_1 &= \int_{x\in\mathbb{R}} |\rho_1(x) - \rho_2(x)| dx = \int_{x\in\mathbb{R}} \left| \sum_{\langle v,v'\rangle\in\widehat{D}_1} (v'-v)\cdot g_v(x) - \sum_{\langle u,u'\rangle\in\widehat{D}_2} (u'-u)\cdot g_u(x) \right| dx \\
&= \int_{x\in\mathbb{R}} \left| \sum_{w=\langle u,u'\rangle\in\widehat{D}_1} \left[(u'-u)\cdot g_u(x) - \gamma^*(w).pers \cdot g_{\gamma^*(w).birth}(x) \right] \right| dx \\
&\le \int_{x\in\mathbb{R}} \sum_{w=\langle u,u'\rangle\in\widehat{D}_1} \left| (u'-u)\cdot g_u(x) - \gamma^*(w).pers \cdot g_{\gamma^*(w).birth}(x) \right| dx \\
&= \sum_{w=\langle u,u'\rangle\in\widehat{D}_1} \int_{x\in\mathbb{R}} \left| w.pers \cdot g_u(x) - \gamma^*(w).pers \cdot g_{\gamma^*(w).birth}(x) \right| dx \\
&\le \sum_{w=\langle u,u'\rangle\in\widehat{D}_1} [|w.pers - \gamma^*(w).pers| + \sqrt{\frac{2}{\pi}} \cdot \min\{w.pers, \gamma^*(w).pers\} \cdot |w.birth - \gamma^*(w).birth|] \\
&\le \sum_{w=\langle u,u'\rangle\in\widehat{D}_1} [\|w - \gamma^*(w)\|_\infty + \sqrt{\frac{2}{\pi}}\Delta \|w - \gamma^*(w)\|_\infty \le (1 + \sqrt{\frac{2}{\pi}}\Delta)\cdot d_{W,1}(D_1, D_2).
\end{aligned}$$

Theorem 0.1 then follows.

References

[1] Cohen-Steiner D, Edelsbrunner H, Harer J. Stability of Persistence Diagrams. Discrete & Computational Geometry. 2007;37(1):103–120.

[2] Chazal F, Cohen-Steiner D, Glisse M, Guibas LJ, Oudot S. Proximity of persistence modules and their diagrams. In: Proc. 25th ACM Sympos. on Comput. Geom.; 2009. p. 237–246.

[3] Chazal F, de Silva V, Glisse M, Oudot S. The structure and stability of persistence modules. CoRR. 2012;abs/1207.3674.

[4] Bauer U, Ge X, Wang Y. Measuring distance bewteen Reeb graphs. In: Proc. 30th SoCG; 2014. p. 464–473.

[5] Dey TK, Shi D, Wang Y. Comparing graphs via persistence distortion. In: Proc. 31rd Annu. Sympos. Comput. Geom. (SoCG); 2015. p. 491–506.

[6] Gromov M. Metric structures for Riemannian and non-Riemannian spaces. volume 152 of *Progress in Mathematics*. Birkhäuser Boston Inc.; 1999.

[7] Mémoli F. On the use of Gromov-Hausdorff Distances for Shape Comparison. In: Symposium on Point Based Graphics; 2007. p. 81–90. Available from: `http://www.eg.org/EG/DL/WS/SPBG/SPBG07/081-090.pdf`.

[8] Cohen-Steiner D, Edelsbrunner H, Harer J, Mileyko Y. Lipschitz Functions Have L_p-Stable Persistence. Foundations of Computational Mathematics. 2010;10(2):127–139. doi:10.1007/s10208-010-9060-6.

[9] Adams H, Chepushtanova S, Emerson T, Hanson E, Kirby M, Motta F, et al.. Persistent Images: A Stable Vector Representation of Persistent Homology; 2015.
