# Supplementary material for: Metrics for comparing neuronal tree shapes based on persistent homology
Source: PLoS One. 2017 Aug 15;12(8):e0182184. doi: 10.1371/journal.pone.0182184 (PMC5557505; doi:10.1371/journal.pone.0182184)
Supplement: S2 File — (PDF) [file pone.0182184.s002.pdf]

Supplement S2 :
Additional Details for Experimental Results

Using Euclidean distance as descriptor function. In Table 1, we provide the number of hits and success-rates for k-nearest neighbor classification based on persistence diagrams (d_P) and persistence-vectors (d_V) using Euclidean distance function as descriptor function. We note that the success-rates are comparable to (but slightly worse than) those obtained from the geodesic distance descriptor function.

Table 1: Leave-one-out cross validation for k-NN classification using Euclidean distance function as descriptor function

	# neurons classified correctly / success-rate for Dataset 1	
# nearest neighbors k	Persist-distance d_P	Persist-vec d_V
1	123 / 0.3555	166 / 0.4798
2	152 / 0.4393	200 / 0.5780
3	164 / 0.4740	221 / 0.6387
4	189 / 0.5462	238 / 0.6879
5	203 / 0.5867	245 / 0.7081

Comparison with L-Measure quantities. We also compare our persistence-based features with specially-designed summaries of neuron morphology contained in L-Measure [1]. This experiment is carried out for Dataset 1. We use only those L-Measure parameters reported in the meta-data associated to each neuron in NeuroMorpho.Org [2], which can be considered as common L-Measure quantities. These parameters are listed in Table 2 – We exclude the parameter "Soma surface " and "Number of stems" as the first one is not reported (i.e, 'N/A') for neurons in Dataset 1, and the second one is the same for all of them. For each individual parameter, we also list the success-rate of k-nearest neighbor classification when the distance between two neurons is computed based on this parameter alone (defined as the absolute difference between its values for the two neurons).

Table 2: Leave-one-out cross validation using each parameter alone for Dataset1

	Results for different number of nearest neighbors				
L-Measure Parameters	1	2	3	4	5
Number of Bifurcations	52 / 0.1503	86 / 0.2486	100 / 0.2890	125 / 0.3613	150 / 0.4335
Number of Branches	45 / 0.1301	81 / 0.2341	107 / 0.3092	127 / 0.3671	150 / 0.4335
Overall Width	19 / 0.0549	38 / 0.1098	54 / 0.1561	66 / 0.1908	80 / 0.2312
Overall Height	52 / 0.1503	92 / 0.2659	105 / 0.3035	118 / 0.3410	133 / 0.3844
Overall Depth	30 / 0.0867	53 / 0.1532	74 / 0.2139	93 / 0.2688	103 / 0.2977
Average Diameter	23 / 0.0665	50 / 0.1445	64 / 0.1850	69 / 0.1994	78 / 0.2254
Total Length	46 / 0.1329	77 / 0.2225	101 / 0.2919	120 / 0.3468	135 / 0.3902
Total Surface	42 / 0.1214	71 / 0.2052	104 / 0.3006	131 / 0.3786	151 / 0.4364
Total Volume	52 / 0.1503	85 / 0.2457	111 / 0.3208	126 / 0.3642	136 / 0.3931
Max Euclidean Distance	51 / 0.1474	84 / 0.2428	117 / 0.3382	147 / 0.4249	168 / 0.4855
Max Path Distance	41 / 0.1185	66 / 0.1908	78 / 0.2254	94 / 0.2717	111 / 0.3208
Max Branch Order	26 / 0.0751	56 / 0.1618	66 / 0.1908	79 / 0.2283	86 / 0.2486
Average Contraction	9 / 0.0260	11 / 0.0318	19 / 0.0549	33 / 0.0954	33 / 0.0954
Total Fragmentation	46 / 0.1329	79 / 0.2283	102 / 0.2948	118 / 0.3410	129 / 0.3728
Partition Asymmetry	13 / 0.0376	29 / 0.0838	45 / 0.1301	60 / 0.1734	58 / 0.1676
Average Rall's Ratio	6 / 0.0173	24 / 0.0694	46 / 0.1329	52 / 0.1503	58 / 0.1676
Avg. Bifurcation angle local	14 / 0.0405	29 / 0.0838	37 / 0.1069	46 / 0.1329	65 / 0.1879
Avg. Bifurcation angle remote	32 / 0.0925	48 / 0.1387	72 / 0.2081	92 / 0.2659	104 / 0.3006
Fractal Dimension	5 / 0.0145	18 / 0.0520	24 / 0.0694	33 / 0.0954	41 / 0.1185

Next, we create a new feature-vector, denoted by L_T, for each neuron T from $\mathcal{S}$ = Dataset 1, where L_T simply consists of all the L-Measure quantities listed in Table 2. To compare two neurons under this feature vectorization, we use normalized L_2-norm between two vectors L_{T_1} and L_{T_2}. In particular, for the i-th parameter p_i, we first compute the mean μ_i and standard deviation σ_i of it from its values over all neurons in $\mathcal{S}$ (Dataset 1); i.e, $\mu_i = \frac{1}{|\mathcal{S}|}\sum_{T\in\mathcal{S}} L_T[i]$ and $\sigma_i = \sqrt{\frac{\sum_{T\in\mathcal{S}}(L_T[i]-\mu_i)^2}{|\mathcal{S}|}}$. Here, $L_T[i]$ stands for the i-th entry of vector L_T, which is the value of this parameter p_i for neuron T. Let ℓ denote the total number of L-Measure quantities we consider (i.e, the dimension of the feature vector L_T). The normalized L_2-distance between two feature vectors L_{T_1} and L_{T_2} is defined as:

$$d_L(T_1, T_2) = \Big[\sum_{i=1}^{\ell}\big(\frac{L_{T_1}[i]-\mu_i}{\sigma_i} - \frac{L_{T_2}[i]-\mu_i}{\sigma_i}\big)^2\Big]^{1/2}.$$

In other words, this is the standard L_2-distance between L_{T_1} and L_{T_2} after each entry is normalized by its respective mean and variance.

The comparison of k-nearest neighbor classification accuracy is reported in Figure 7 (B) in the main text of the submission.

References

[1] Scorcioni R, Polavaram S, Ascoli GA. L-Measure: A web-accessible tool for the analysis, comparison and search of digital reconstructions of neuronal morphologies. Nature Protocols. 2008;3(5):866–876.

[2] Ascoli GA, Dohohue DE, Halavi M. NeuroMorpho.Org: A central resource for neuronal morphologies. J Neurosci. 2007;27(35):9247–51.
